# The Extreme Conditions Beamline P02.2 and the Extreme Conditions Science Infrastructure at PETRA III

**DOI:** 10.1107/S1600577515005937

**Published:** 2015-06-19

**Authors:** H.-P. Liermann, Z. Konôpková, W. Morgenroth, K. Glazyrin, J. Bednarčik, E. E. McBride, S. Petitgirard, J. T. Delitz, M. Wendt, Y. Bican, A. Ehnes, I. Schwark, A. Rothkirch, M. Tischer, J. Heuer, H. Schulte-Schrepping, T. Kracht, H. Franz

**Affiliations:** a Deutsches Elektronen-Synchrotron (DESY), Hamburg, Germany; b Department of Crystallography, University of Frankfurt, Frankfurt, Germany; c Bayrisches Geoinstitut, University of Bayreuth, Bayreuth, Germany

**Keywords:** extreme conditions, high-pressure, high-temperature, low temperature, diamond anvil cell, micro-diffraction, time-resolved diffraction, radial diffraction, laser heating, resistive heating, cryostat, PDF, high energies

## Abstract

A detailed description is presented of the Extreme Conditions Beamline P02.2 for micro X-ray diffraction studies of matter at simultaneous high pressure and high/low temperatures at PETRA III, in Hamburg, Germany. This includes performance of the X-ray optics and instrumental resolution as well as an overview of the different sample environments available for high-pressure studies in the diamond anvil cell. Particularly emphasized are the high-brilliance and high-energy X-ray diffraction capabilities of the beamline in conjunction with the use of fast area detectors to conduct time-resolved compression studies in the millisecond time regime. Finally, the current capability of the Extreme Conditions Science Infrastructure to support high-pressure research at the Extreme Conditions Beamline and other PETRA III beamlines is described.

## Abstract 2

Performance description of the Extreme Conditions Beamline (ECB, P02.2) at PETRA III that is optimized for micro-diffraction at simultaneous high pressure and high and low temperatures created in different diamond anvil cells environments. Additional information of the capabilities of the Extreme Conditions Science Infrastructure for DAC work is provided.

## 1. Introduction   

Diamond anvil cells (DACs) have been used for many years to study the properties of materials at high pressures and simultaneous high/low temperatures in order to explore, for example, elastic properties of materials important to geophysics as well as high-pressure physics (*e.g.* Hemley & Mao, 2002 ▸; Duffy, 2005 ▸; Hemley *et al.*, 2005 ▸) and to systematically explore the crystal chemistry of materials ranging from simple elements to more complex materials, for example oxides, nitrides, carbides, *etc*. (*e.g.* McMillan, 2004 ▸; Schettino *et al.*, 2005 ▸). Many of these experiments heavily rely on structural analysis using powder and single-crystal X-ray diffraction patterns. While laboratory X-ray sources can provide some information at lower pressures because of the lack of photon flux and micrometer beam size at low divergence (*e.g.* Dubrovinsky *et al.*, 2001 ▸; Angel *et al.*, 2007 ▸), only the use of very brilliant second- and third-generation synchrotron-radiation-based hard X-ray sources have enabled multi-megabar experiments at temperatures from the single digit to several thousands of Kelvin. In spite of these advances, most experiments have been and are still conducted under static conditions. Only in the last decade has the high-pressure community seen the emergence of more and more diffraction and spectroscopic studies that aim at understanding the dynamic processes that can be created within the dynamic driven DAC (Anzellini *et al.*, 2013 ▸; Goncharov *et al.*, 2010 ▸; Evans *et al.*, 2007 ▸). Such experiments demand higher brilliance, faster detectors and new sample environments that can create dynamic conditions. Thus, it is justified to design and build a ‘Hard X-ray Diffraction’ beamline at a high-energy third-generation light source such as PETRA III, DESY, Hamburg, that can meet these new challenges.

Here we present the design, performance and capabilities of the Extreme Conditions Beamline (ECB; P02.2) that started its operation in March 2011 at PETRA III. The goal of the ECB is to provide dedicated tools to conduct powder and single-crystal X-ray diffraction experiments at simultaneous high pressure (1 bar to 4 Mbar) and low/high temperatures (2–5000 K) within different types of DACs. Particular focus of the beamline is the development and implementation of time-resolved diffraction capabilities, such as single-exposure diffraction experiments in the millisecond regime. Finally, we describe the Extreme Conditions Science Infrastructure and its capabilities that may be used for extreme conditions research at the ECB in particular and DESY in general.

## 2. Storage ring PETRA III and location of the ECB   

**Figure 1:**
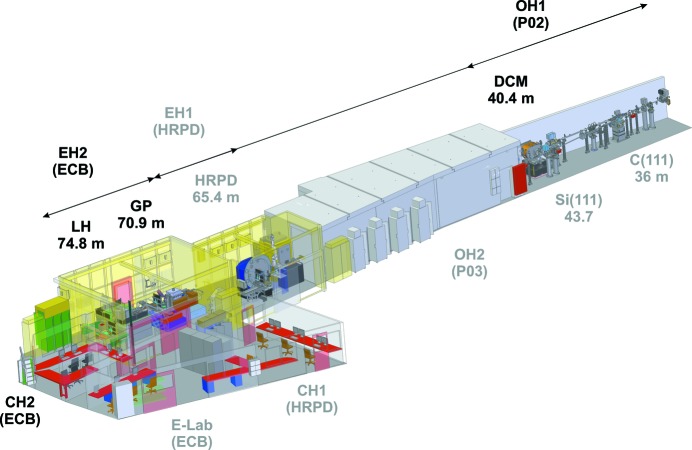
View of sector 2 indicating the location of P02 that consists of the joint P02 optics hutch (OH1), the HRPD branch (EH1) and the ECB branch (EH2) with the laser heating (LH) and the general purpose (GP) experiments. P03, the ‘Micro- and Nanofocus X-ray Scattering’ (MiNaXS) beamline for small-angle scattering is located downstream of the ‘Hard X-ray Diffraction Beamline’, while its optics hutch is positioned between the optics hutch OH1 of P02 and the experimental hutch EH1 of P02.1, the HRPD beamline.

Extreme conditions research using DACs is heavily dependent on access to third-generation light sources since samples become very small at very high pressures (*e.g.* Tateno *et al.*, 2010 ▸). The increased brilliance of newer third-generation light sources results in more effective focusing, even at high energies, and enables time-resolved single-exposure diffraction experiments on the milliseconds timescale, as well as pump–probe (stroboscopic) measurements down to hundreds of picoseconds. PETRA III is a good example of such a new third-generation light source, utilizing a highly focused electron beam with very small emittance, creating a very small and intense source of X-rays with low divergence and high degree of coherence. The relevant storage ring parameters for PETRA III are listed in Table 1 ▸. In sector 2, a pair of 2 m-long undulators is placed in a 5 m-long straight section with a canted separation of 5 mrad providing space for beamlines P02 (Hard X-ray Diffraction Beamline, Fig. 1 ▸) and P03 (‘Micro- and Nanofocus X-ray Scattering beamline’, MiNaXS). In order to increase the separation between the two beamlines further, the MiNaXS beamline uses a large-offset monochromator (Roth *et al.*, 2011 ▸; Buffet *et al.*, 2012 ▸) to reflect the X-ray beam downward by 500 mm offering a large separation between the transfer pipe of P03 and the sample position of the main line of P02. Furthermore, beamline P02 consists of two parallel operating branches, P02.1 (HRPD, ‘High Resolution Powder Diffraction’ beamline) and P02.2 (ECB). This is possible because the HRPD beamline is branched off by 1.5 m through a diamond (111) combined with a silicon (111) monochromator crystal in Laue geometry to the horizontally displaced side-station (Dippel *et al.*, 2015 ▸). Since both instruments offer 100% beam time during PETRA III operation, X-rays that are transmitted through the diamond Laue crystal along the straight line towards the ECB are fixed to energies of the third, fifth, seventh and ninth harmonic at 25.6, 42.7, 60.0 and 77.1 keV, respectively.

## 3. Outline of the ECB   

**Figure 2:**
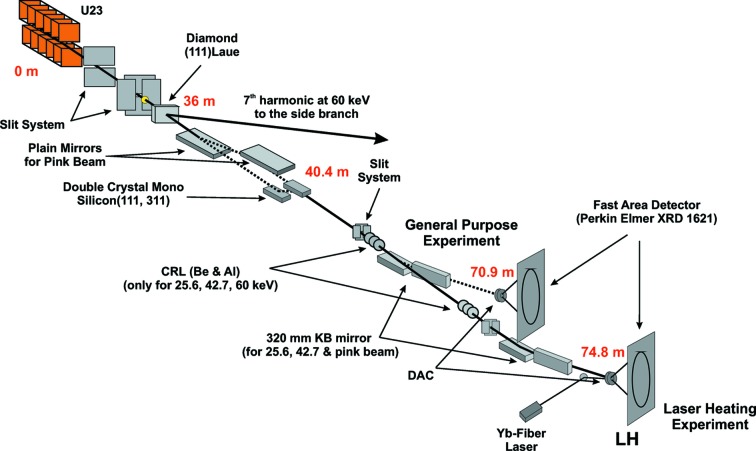
Optical train of the Hard X-ray Diffraction Beamline P02 at PETRA III. Important components are the pair of plain mirrors for pink-beam transfer (in commissioning), double-crystal monochromator (DCM), compound reflective lenses (CRL) for relative large focus [∼8 µm (H) × 2 µm (V) FWHM] with low divergence, and Kirkpatrick–Baez (KB) mirror system for small focus [∼2 µm (H) × 2 µm (V) FWHM].

The optical train of the ECB is displayed in Fig. 2 ▸. Major optical components are:

(i) the high-energy undulator, type U23, with white-beam slits and carbon/copper filters in the front end;

(ii) the standard PETRA III high-heat-load double-crystal monochromator that is preceded by the diamond Laue monochromator of the side-station;

(iii) the two focusing devices, a 320 mm Kirkpatrick–Baez mirror system and a compound refractive lens system;

(iv) the pair of mirrors in front of the high-heat-load monochromator to enable pink-beam diffraction (still under commissioning).

### 3.1. Insertion device and front-end   

**Figure 3:**
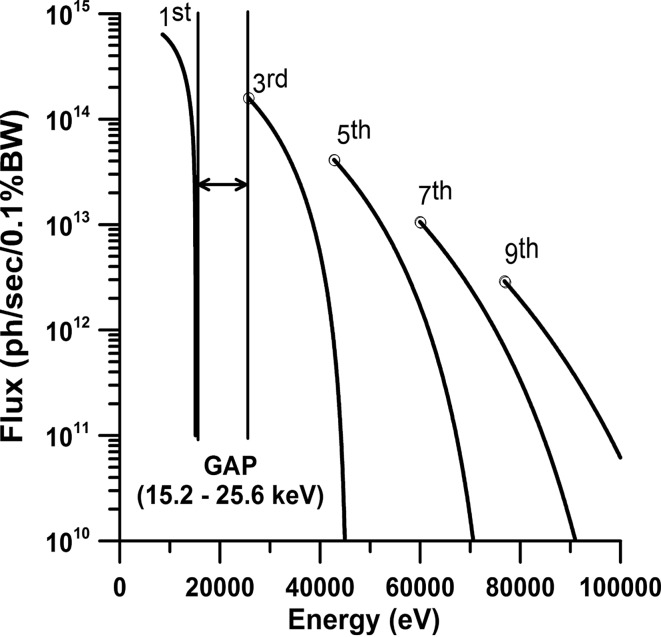
Flux (photons s^−1^ 0.1% BW^−1^) as a function of energy for the undulator harmonics of the undulator U23 calculated using *SPECTRA 9.0.2* (Tanaka & Kitamura, 2001 ▸) at 29.3 m from the source within a 1 mm × 1 mm (35.7 µrad × 35.7 µrad) aperture. This corresponds to the theoretical available flux after the second high-heat-load slit system of the beamline. In order to protect the first monochromator crystal of the HRPD beamline branch the slits are never opened larger than 1 mm × 1 mm. The calculated flux values are used as a starting value for the ray-tracing calculation in *SHADOW* after the first high-heat-load slit system. The energy spectrum of U23 has a gap between 15.2 keV of the first and 25.6 keV of the third harmonic (minimum gap of 10 mm). Fixed energies of 25.6, 42.7, 60.0 and 77.1 keV used at the beamline are highlighted by the open circles at the end of the third, fifth, seventh and ninth harmonic and correspond to maximum flux reachable at the minimum gap of ∼10 mm.

P02 is powered by a U23 undulator optimized for high energies and currently operated in high-β configuration. Parameters in Table 1 ▸ were used to calculate the flux of the X-ray beam at 29.3 m from the source in a 1 mm^2^ area (Fig. 3 ▸). In comparison with other third-generation light sources that operate at 6–8 GeV, PETRA III displays the lowest horizontal emittance, 1 nmrad. As a consequence the source size and divergence of the X-ray beam created in the undulator is decreased resulting in a higher brilliance as compared with similar high-energy beamlines. The undulator is usually set to its minimum gap of ∼10 mm in order to tune the seventh harmonic to ∼60 keV and provide the maximum amount of photons to both branches of P02. The undulator is followed by two pairs of water-cooled high-heat-load slit systems. The first pair of slits is located at 19.6 m from the source. This slit system is set to 1 mm in the vertical direction to cut the low-energy part of the undulator radiation and to reduce the heat load on the subsequent devices. The second slit system located at 29.3 m from the source is usually set to 1 mm × 1 mm. Downstream of the first and second slit systems we placed a filter system with movable filters, offering a 1 mm glassy carbon as well as a 0.3 mm CVD diamond filter that is coated with 0.05 mm of copper (Hahn, 2009 ▸). The HRPD beamline always operates with the CVD diamond copper filter in place and the slit system set to no larger than 1 mm × 1 mm in order to reduce the heat load on the diamond (111) Laue monochromator crystal and to create optimal operation conditions for the side-station. This filter has a minor effect on the performance of the straight branch of the beamline because only a factor two of the flux of the third and a factor 0.1 of the fifth harmonic are absorbed. Despite this flux reduction, the ECB beamline performance at 25.6 keV is still competitive because (*a*) the increased brilliance enables the Kirkpatrick–Baez (KB) mirrors to collect a large portion of the incident beam, and (*b*) the undulator is set to its minimum gap providing maximum photons in this energy range. At an energy of 42.7 keV the provided flux is superior in comparison with other extreme condition beamlines. Furthermore, Fig. 3 ▸ illustrates that the ECB can be operated at energies as high as 94.3 keV using the 11th harmonic and that there is an energy gap between 15.2 and 25.6 keV located between the first and third harmonic. Neither the insertion of the absorption filter nor the diamond (111) Laue monochromator crystal had significant effect on the shape of the incident X-ray beam based on optical inspection of the beam structures in the fluorescence image of the unfocused X-rays seen on a La crystal located at the sample position.

### 3.2. Optics   

The high-heat-load double-crystal monochromator and the pair of mirrors for pink-beam delivery are located in the optics hutch of P02, whereas the focusing devices are positioned in the experimental hutch near the sample position. Upstream from the focusing systems, two slit systems (JJ X-ray A/S, Kgs. Lyngby, Denmark) shape the incident beam to the maximum beam acceptance of the optical components. The flux of the incident beam and the pre-shaped beam is monitored with ion chambers (Advanced Design Concepts, Pewaukee, WI, USA).

#### 3.2.1. Double-crystal monochromator   

**Figure 4:**
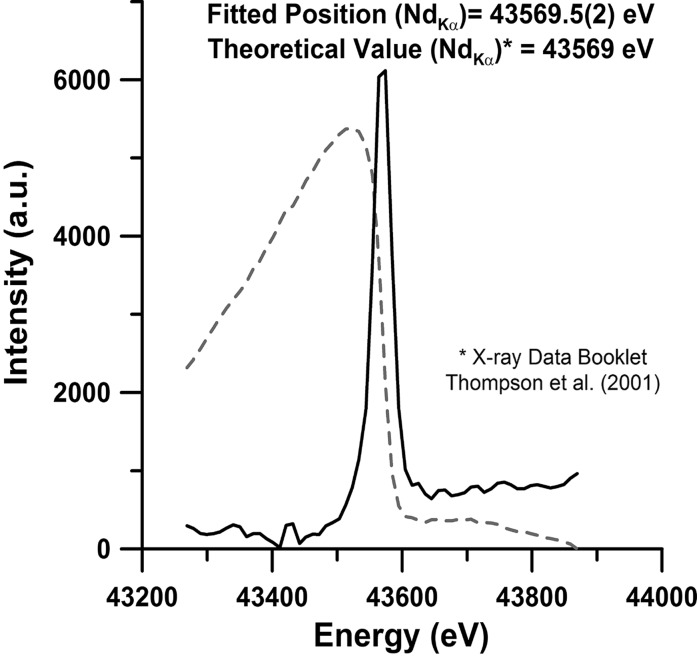
*K*-edge scan from 0.1 mm-thick Nd foil (*K* = 43569 eV). The dotted line represents the actual normalized edge scan and the black solid line the differentiated profile. ▸

**Figure 5:**
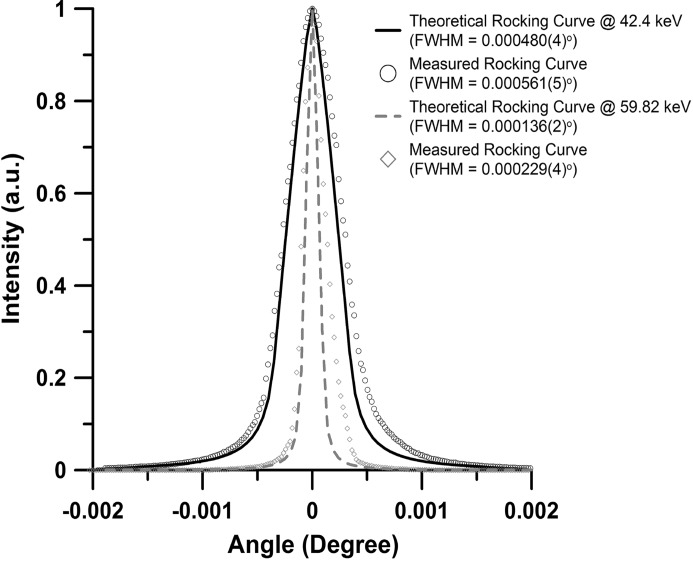
Rocking curves of the Si(111) crystal at 42.4 keV and Si(311) crystals at 59.82 keV of the DCM. The rocking curves (open circles and diamonds) were collected by scanning the second crystal [Si(111) or Si(311)] through the beam of the first crystal. The theoretical rocking curve is displayed as a solid and dashed line. The FWHM of the rocking curve can be used to calculate the maximum value for the energy resolution. Real values of Δ*E*/*E* may be smaller.

A PETRA III standard double-crystal monochromator (DCM) (FMB Oxford Ltd., Osney Mead, Oxford, UK) with a Si(111) and a Si(311) crystal pair has been installed at the ECB (Schulte-Schrepping, 2009 ▸). It provides monochromatic X-rays with an energy resolution of Δ*E*/*E* = ∼2 × 10^−4^ in the range 8.5–54 keV using the Si(111) crystal and Δ*E*/*E* = ∼5 × 10^−5^ in the range 8.5–100 keV for the Si(311) at a fixed offset of 23 mm to satisfy radiation safety requirements. The lower-energy bound of the monochromator is constrained by the smallest gap setting of the undulator. However, the design of the monochromator allows it to operate at energies down to 2.5 keV. The different energies at which the ECB is running are calibrated with *K*-edge scans on standard foils of Sb (*K* = 30.491 keV; EXAFS Materials) for ∼25.6 keV, 0.1 mm Nb (*K* = 43.569 keV) for ∼42.7 keV (Fig. 4 ▸) and 0.05 mm-thick foils of Ta (*K* = 67.416 keV) and Pt (*K* = 78.395 keV; Goodfellow GmbH, Bad Nauheim, Germany) for energies of ∼60.0 and ∼77.1 keV, respectively. After performing the calibration with Nd and Ta edge scans, we found an upper bound of Δ*E*/*E* of the monochromator (see below) set to 42.4 and 59.82 keV to be 2.1 × 10^−4^ for Si(111) and 6.3 × 10^−5^ for Si(311), respectively. The theoretical and measured FWHMs of the rocking curve of the first Si(111) crystal of the DCM at 42.4 keV as well as the Si(311) crystal at 59.82 and 76.95 keV are listed in Table 2 ▸ and displayed for 42.4 and 59.82 keV in Fig. 5 ▸. The Δ*E*/*E* values calculated from the FWHM of the rocking curve represent an upper bound for the energy resolution.

#### 3.2.2. Focusing   

**Figure 6:**
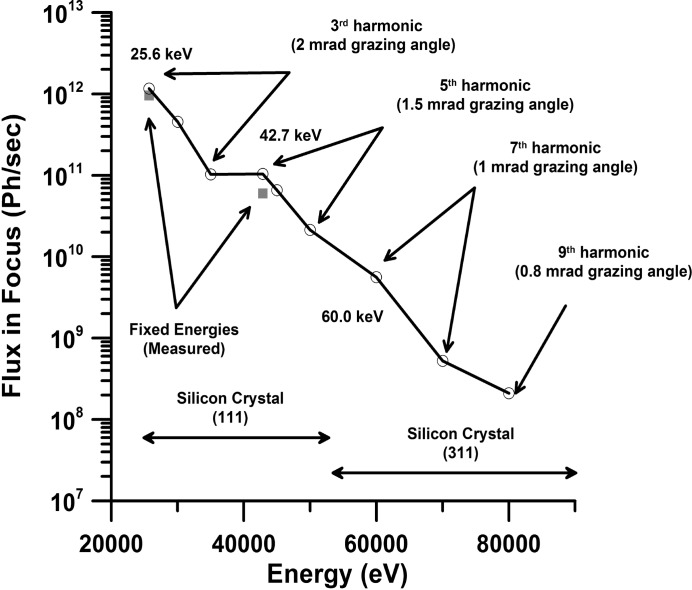
Flux calculated (open circles) within *SHADOW* (Welnak *et al.*, 1992 ▸) for the focal spot of the KB mirror system as a function of energy. Superimposed are the flux values measured (solid squares) with a calibrated diode from the APS within the focal spot. Note that the flux was measured with a 40 µm pinhole while the calculated flux is without a pinhole. Indicated are also the harmonics of the undulator U23 used for the calculation, the crystal in the high-heat-load monochromator as well as the grazing-incident angle applied to the KB mirror system.

The ECB offers two types of focusing: (*a*) compound refractive lenses (CRLs) consisting of Be or Al for diffraction experiments with fixed energies of 25.6, 42.7 (Be), 60.0 and 77.1 keV (Al); (*b*) KB mirror systems for diffraction experiments with a monochromatic beam of 25.6, 42.7 keV (Fig. 6 ▸) and pink beam. Average X-ray beam parameters obtained using the different focusing devices and energies are summarized in Table 3 ▸. The CRLs have been used to focus the beam to a larger focal size while maintaining a smaller divergence. In contrast, the KB mirror system is dedicated to experiments requiring the smallest possible focal spot at 25.6 and 42.7 keV with maximum flux but exhibit a large divergence that is limiting the resolution of the beamline setup (see §3.4.3). To change from the CRL to the KB mirror system, the upstream positioned CRLs are taken out of the beam path and the mirrors of the KB system are driven to their original position and *vice versa*. Within the standard errors the focus of either system remains virtually unchanged after switching.

In the following we will use a right-handed coordinate system that describes the downstream X-ray beam as the positive *x*-direction and the direction horizontal and vertical to the downstream X-ray beam as the *y*- and *z*-direction, respectively.

**Figure 7:**
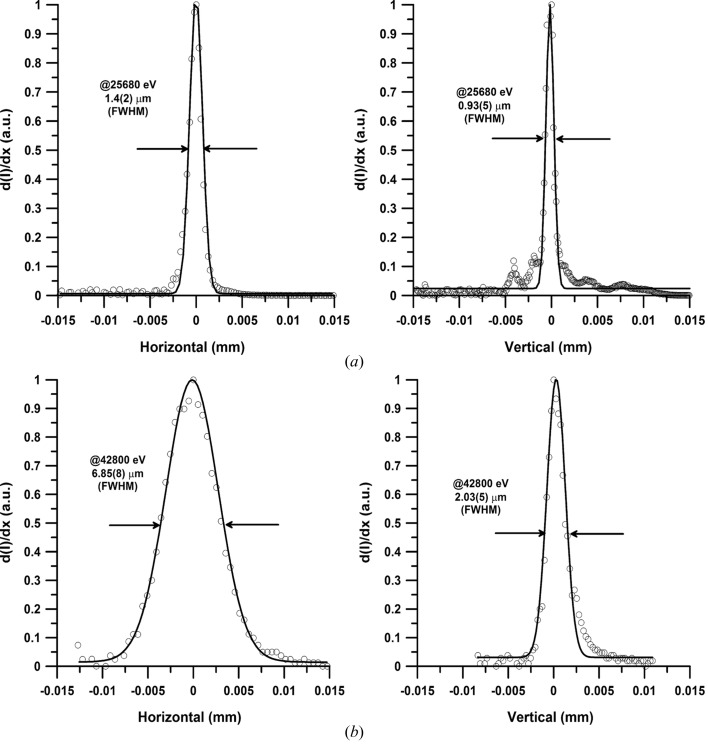
(*a*) Focus achieved at the ECB using the KB mirror at 25.68 keV. (*b*) Focus achieved employing CRLs at 42.8 keV.

*CRLs.* CRLs are designed to focus upstream from the KB mirror system. The expected focal spot size at the sample position was chosen based on prior experience of other high-pressure beamlines [*e.g.* HPCAT at the Advanced Photon Source (APS) and ID27, ID9a at the European Synchrotron Radiation Facility (ESRF)]. Experiments at these facilities often need a larger focal spot for very light or weakly scattering compounds such as organic or non-crystalline materials in order to increase the scattering volume. Thus, we aimed at a focus of 5–6 µm in the horizontal direction. Using the *CRCALC* software (Lengeler *et al.*, 2005 ▸) we estimated that we can achieve a focal spot of around 5 µm in the horizontal and 0.2 µm in the vertical when using 40 (112) CRL lenses consisting of beryllium with a radius of 50 µm, corresponding to an effective beam acceptance of 370 (313) µm at 25.6 (42.7) keV. The focal length of the above lens packages was calculated to be 1220 mm, resulting in a demagnification of 70000 mm/1220 mm = 57.4. Lenses are positioned in a V-groove lens holder (Lengeler *et al.*, 2005 ▸) and are aligned with respect to the incident beam using a motorized positioning system with five degrees of freedom. Simple scans of the lens holder in the five directions provided an average focal spot of ∼8 µm (H) × 2 µm (V) FWHM at both 25.6 and 42.7 keV (Table 3 ▸, Fig. 7 ▸). The focal spot size was determined with a 5 mm-diameter polished round edge of WC located at the sample position. The calculated divergence of the beam is ∼0.31 (0.25) mrad in both directions at 25.6 (42.7) keV. Using a calibrated diode (Zhao, 2014 ▸) we determined the flux in the focal spot to be approximately 2 × 10^11^ (25.6 keV) and 5 × 10^10^ photons s^−1^ (42.7 keV), normalized to 100 mA stored beam, with a clean-up pinhole of 40 µm (positioned at ∼50 mm upstream from the sample) and taking into account the air absorption at the respective energies. Deviations from the theoretically calculated focal spot size and flux (Fig. 6 ▸) may be attributed to the fact that the calculations in *CRCALC* do not take into account the Rayleigh length of the CRLs at the given energies.

*KB mirrors.* KB mirror systems were designed and manufactured by Instrument Design Technology (IDT Ltd., Widnes, UK) in collaboration with P. Eng (University of Chicago) using the beamline parameters described above (Table 1 ▸). The task was to achieve a focal spot size close to 1 µm^2^ while maintaining a long enough focal length to accommodate a laser heating, cryostat and other experimental setups. Calculations indicated that a beam focus of about 3 µm (H) × 3 µm (V) FWHM would be possible with a pair of optimized trapezoidal mirrors assuming a slope error of 1 µrad and a demagnification of 71000 mm/340 mm = 208 in the horizontal and 71000 mm/680 mm = 104 in the vertical direction. Platinum coating on the mirrors supports a maximum grazing incident angle of 2 mrad (25.6 keV) and 1.5 mrad (42.7 keV) and an acceptance of 530 µm (H) and 420 µm (V), respectively, of the incident beam assuming a footprint of 280 mm. The divergence of the beam focused by the KB mirrors is relatively large with 1.2 (H) and 0.63 (V) mrad as expected for demagnifications of 208 and 104 at a distance of 71 m from the source. These values are larger than the values calculated for the CRL systems. In order to be able to serve two experiments [laser heating (LH) and general purpose (GP) experiments separated by 4 m along the beam; see below] with the same focusing capability, two identical mirror systems were purchased. The differences in the demagnification due to the changed distance between mirrors and source have a minor effect on the focal spot of the LH *versus* the GP experiment, so that we were able to use identically shaped mirrors. In contrast to earlier systems built by IDT we decided to place the mirror bender in a vacuum chamber (10^−5^–10^−7^ mbar) instead of flushing it with He. After three years of operation we have not observed any deposition of contaminants on the mirrors.

Fig. 7 ▸ depicts the best focus reached with the 320 mm-long KB system at the laser heating experiment. Focus size was determined with a 5 mm WC round edge. It was better than expected compared with the focus size calculated with *SHADOW* using the slope error estimated from metrology measurements conducted at the APS. The reason for the improved focus probably originates from the dynamic bending of the trapezoidal mirrors that improves the overall slope error and consequently the focus. The flux values are in good agreement with the values calculated using the *SHADOW* program (Welnak *et al.*, 1992 ▸; Fig. 6 ▸).

### 3.3. Detection system   

Detection of powder and single-crystal diffraction images can be accomplished by different area detectors that can be installed depending on the requirements of the experiment. Since one of the specializations of this beamline is the support and development of time-resolved diffraction capabilities (see §3.6) we have opted to install area detectors with a fast readout time and/or accumulating capabilities. For high-energy diffraction experiments (>20 keV) we have implemented the fast area detector XRD 1621 (Perkin­Elmer) and the ‘standard’ Mar345 image plate (marXperts, formerly Marresearch). The XRD 1621 consists of a CsI scintillator bonded to an amorphous silicon chip. The detector offers a pixel size of 200 µm × 200 µm and a maximum readout speed of 15 frames per second (fps) at maximum resolution. The speed of the detector can be increased to a maximum of 30 fps when binning pixels to clusters of 400 µm × 400 µm. We use the software *QXRD* (Jennings, 2014 ▸) for controlling the detector. *QXRD* offers the possibility to subtract background as well as the summation of multiple images at the readout speed of the detector. Furthermore, the program is capable of performing on-the-fly data integration for online data analysis as well as data reduction [radially integrating two-dimensional X-ray diffraction (XRD) patterns to one-dimensional intensity profiles]. The latter is an essential tool for conducting and evaluating fast time-resolved diffraction experiments. We have estimated the point spread function (PSF) of the XRD 1621 by measuring the line spread function (LSF) of the detector by covering half of a well defined and unfocused direct beam at 60 keV with a tungsten round edge and determined it to be 1.3 pixels (FWHM), in agreement with LSF measurements of 1.1 pixels (FWHM) on the equivalent detector distributed by General Electric (Lee *et al.*, 2008 ▸). In comparison, the Mar345 offers a PSF of 2.8 pixels at 80 keV (Daniels & Drakopoulos, 2009 ▸) at its maximum resolution (100 µm × 100 µm pixel size).

For very short single-exposure experiments we are currently using the PILATUS 300K or 1M detectors available through the DESY detector pool. At both experiments (LH and GP experiment, see §3.4) two detectors can be mounted simultaneously; while the XRD 1621 is permanently installed, the Mar345 can be replaced by the PILATUS detectors. All detectors are placed on a translation system that permits sample-to-detector distances (SDDs) of 350–1100 mm for the XRD 1621 at the LH experiment (350–950 mm at the GP experiment) and large lateral movement so that the detectors may be placed centered or off-centered to the beam, *e.g.* to increase/optimize the access to reciprocal space. At the standard setting of SDD = 400 mm the XRD 1621 is able to collect diffraction through a DAC opening angle of ±45°. Tilt and yaw of the XRD 1621 can be adjusted through a cradle and rotation beneath the detector to align it perpendicular to the incident beam. Such capability is particularly useful for highly precise single-crystal diffraction experiments. The Mar345 and PILATUS detectors can only be adjusted in yaw. Of particular interest for all detectors is the yaw rotation in conjunction with the lateral and parallel detector movements that may be used to create a virtual detector movement on a circle, so that the center of the detector has always the same SDD.

### 3.4. Experimental settings and diffraction techniques   

Standard sample environments for extreme conditions research found at third-generation light sources consist of dedicated laser heating systems for the DAC as well as support for resistive heated and cryogenically cooled DAC experiments. In order to optimize operation we have spatially separated LH DAC setups from all other GP DAC related sample environments as described below.

#### 3.4.1. LH experiment   

LH systems for the DAC were developed in collaboration with the Mineralogy Section of the Department of Geosciences at the University of Frankfurt (PI: B. Winkler). Details and capabilities of the system will be described elsewhere. However, for completeness we will provide a short overview of the systems. The experimental setup is located in the downstream part of the ECB hutch to minimize time-consuming setup changes, *i.e.* the laser heating configuration and optimized focusing with the KB mirrors will not be changed for operation of bulky equipment. Laser heating optics are placed on 59 mm-thick optical breadboards that are attached to a massive granite support cemented to the base plate of the PETRA III experimental hall. The granite support was dimensioned in such a way that the height of the optical path of the LH system amounts to ∼120 mm from the top of the breadboard. X-ray optics are placed in a recess of the granite support and encapsulated by lead shielding to reduce parasitic scattering. The sample positioning stack is placed on the same granite as the X-ray optics to achieve maximum stability. Details of the sample positioning are described in the next section. Laser heating can be accomplished by guiding the laser through the diamonds at an angle of ∼24° (off-axis) or parallel (on-axis) to the X-ray beam and the compression axis of the DAC. Off-axis heating is accomplished through a 100 W Yb-fiber laser that is located on the outboard side of the experimental setup. The laser is split through a cube splitter and the power of each branch controlled through half-wave plates before focusing on the sample from the upstream and downstream sides for double-sided laser heating. Temperatures of the sample heated by a 5–30 µm laser spot are estimated using pyrometry of the light emitted from the hot sample through a dedicated beam path parallel to the compression axis of the DAC. The optical path terminates in a 303 mm Czerny–Turner spectrograph (Andor Technology, model Shamrock 303) with a fast (2 ns readout time) and gated iStar 312T CCD. The first optical component of the pyrometric beam path is a 45° mirror, requiring that the mirrors have to be either very thin and/or consist of amorphous material in order to permit the incident X-ray beam (upstream) or the diffracted X-rays (downstream) to pass. Alternatively, one may retract the downstream mirror to obtain a clear and uncontaminated diffraction image, with the consequence of not being able to monitor temperatures on the downstream side. The pyrometric beam path may also be used for viewing and aligning the sample to the X-ray beam through very sensitive CCD cameras (ProsilicaG125B ASG). In the case of ‘on-axis’ heating the laser light originates from a 200 W Yb-fiber laser that is located on the inboard side of the experimental setup. The laser is split and the heating power of each branch can be adjusted *via* half-wave plates similar to the off-axis laser heating setup. However, in contrast to the off-axis laser heating setup, the lasers are introduced to the pyrometric observation beam path and guided towards the sample through the two 45° mirrors at the end of the pyrometric optical path. The placement of the mirrors in the beam may potentially contaminate the diffraction image. However, this configuration requires a much smaller opening angle of ±10° in the seat of the DAC resulting in a much better support of the diamonds and thus increases the ability to generate very high pressures. On-axis heating may also be used when conducting pulsed or flash laser heating that will suffer from possible distortions of the laser beam path in heavily stressed diamond anvils when using the off-axis laser heating geometry. Both setups have been frequently used for synthesis studies (Scheler *et al.*, 2013 ▸; Zhang *et al.*, 2013 ▸), to determine high-pressure and temperature phase transitions (Andrault *et al.*, 2014 ▸), and pair distribution function studies (Sanloup *et al.*, 2013 ▸; Rademacher *et al.*, 2014 ▸).

#### 3.4.2. GP experiment   

The GP experiment offers a staging area for sample environments that cannot be accommodated at the LH experiment, such as bulky resistive heated DACs for high-pressure experiments at moderate temperatures (up to 2000 K), or DACs that are placed in a cryostat for high-pressure experiments at low temperatures (currently down to 45 K). The GP experiment is conceptually designed to accept various sample stacks/experimental configurations, *i.e.* the sample alignment system sits on a large three-point kinematic mount that can be easily exchanged. This versatility offers the advantage of using a very accurate sample stack for small and medium loads, *e.g.* required for one rotation axis single-crystal and powder diffraction experiments in the DAC, or light resistive heated/cryogenically cooled DACs. At the same time, heavier sample alignments and support systems, with less alignment accuracy, required for experimental setups such as a four-circle diffractometer and a Paris–Edinburgh press setup can be installed using the kinematic mount.

The standard sample stack used at the LH and GP systems conforms to the typical motorized high-pressure DAC alignment systems, *i.e.* the sample can be positioned in the center of the rotation (omega) through two horizontal translations along and perpendicular to the incident X-ray beam (cenx and ceny) located on top of the omega rotation. Alignment of the rotation center to the X-ray beam is achieved with an additional horizontal translation (SamY) located perpendicular to the X-ray beam, below the omega rotation. The stack is completed with a vertical translation (SamZ) below the omega rotation and an additional horizontal translation parallel to the beam (SamX). Because the X-ray beam size produced by the KB mirror is near or below ∼2 µm (H) × 2 µm (V), we required a positioning system that can offer a step size of 50–100 nm. This was achieved through the usage of a vertical positioner from Micos (NPE-200) and cross roller translations from Huber (5102.15). Normally the stack is operated in a mode without active position feedback; however, all translations and rotations are equipped with encoders that can be used if higher accuracy is required. This high position accuracy ensures adequate characterization of a very small X-ray beam *via* the WC round edge scan as well as positioning and characterization of very small samples holes as common in DACs that are compressed to multi-megabar.

Inboard of the sample stack we have installed an online ruby fluorescence measurement system that may also be used as an online microscope for sample alignment. The system is similar to the offline ruby fluorescence system (see §4.2.1, Fig. 14) with the exception that we use a 457 nm excitation laser with a 100 mW (Single-Frequency DPSS Laser System, LSS-0457-WSO-00100-01, Laser Glow Technologies) which is Raman capable. In addition, the microscope looks at the sample at an angle of 90°, parallel to the X-ray beam. The system is currently operated in ruby fluorescence mode, but awaits an upgrade to a full Raman capable system.

#### 3.4.3. Powder diffraction and instrumental resolution function   

**Figure 8:**
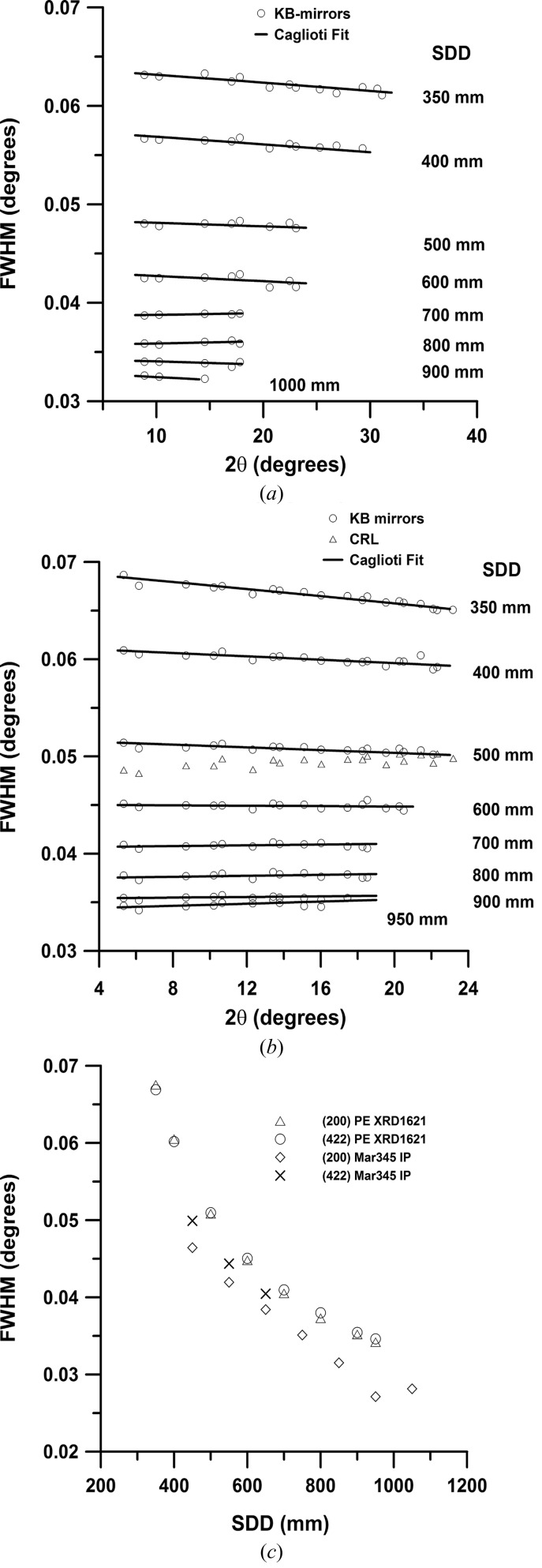
Instrumental resolution function (IRF) measured for a 2 µm × 2 µm KB mirror focused beam collected on an XRD 1621 from PerkinElmer at (*a*) 25.638 keV and (*b*) 42.720 keV as a function of sample-to-detector distance (SDD) using the CeO_2_ powder diffraction standard from NIST (674b) loosely filled into an empty gasket hole with a thickness of 0.03 mm. (*c*) Comparison of the IRF determined from the PerkinElmer XRD1621 and the Mar345 image plate as a function of different SDDs for the (200) and (422) reflections at an energy of 42.72 keV.

Powder diffraction is the standard technique at all high-pressure beamlines. In order to determine the quality of powder diffraction patterns that the KB mirror or CRL focused beam creates and that are collected on the different area detectors, we measured the instrumental resolution function (IRF) at different SDDs on the XRD 1621 flat-panel detector and the Mar345 image plate. Diffraction patterns of a CeO_2_ standard (674b from National Institute of Standards) placed loosely in a gasket hole without diamonds were collected at fixed energies of 25.638 and 42.720 keV and SDD in the range 350–950 mm (450–1050 mm for the Mar345). The sample was rotated ±5° to improve the grain statistics and to suppress inhomogeneous powder rings originating from the mismatch of focus size and crystallite size. The SDD, tilt and rotation of the detector were calibrated in *Fit2d* (Hammersley *et al.*, 1996 ▸; Hammersley, 1997 ▸) and two-dimensional images were azimuthally integrated within. Diffraction peaks of the one-dimensional pattern were individually refined within the program *PeakFit* using a pseudo-Voigt profile function. Obtained FWHMs of the diffraction peaks as a function of 2θ and SDD are plotted in Fig. 8 ▸. The diffraction patterns originated from the 2 µm (H) × 2 µm (V) X-ray beam of the KB mirrors collected on XRD 1621. For comparison we also plot the FWHMs of the diffraction peaks from a pattern collected at a SDD of 500 mm created with a CRL focused beam [8 µm (H) × 3 µm (V)] at 42.709 keV. To assess the quality of the diffraction patterns collected on the XRD 1621 detector we plot in Fig. 8(*c*) ▸ the FWHM of the diffraction peak of selected reflections of CeO_2_ [(200) and (422)] as a function of SDD at 42.720 keV. Finally, we fitted for each SDD a Caglioti function [Caglioti *et al.*, 1958 ▸; FWHM(θ) = (*U*tan^2^θ + *V*tanθ + *W*)^1/2^] to the IRF data. The parameters *U*, *V* and *W* are listed in the supporting information. For the refinement, the value of *U* was fixed to zero since refinements yielded a very small but negative number, *i.e.* the 2θ range covered by the diffraction pattern was too small to constrain *U*tan^2^θ adequately. In addition, we also performed selected Rietveld refinements in *FullProf* to verify that the parameters of the Caglioti function were determined correctly. The fit of the parameters was identical within the errors of both refinements.

Figs. 8(*a*) and 8(*b*) ▸ show that the slope of the FWHM of the diffraction peaks is rather flat and in fact slightly decreases with increasing 2θ at very short SDD. Normally one would expect an increase of the FWHM with increasing 2θ because the theoretically calculated FWHM (Table 4 ▸) should be controlled by the divergence originating from the KB mirror system of 1.23 mrad (H) and 0.63 mrad (V). However, there is a competing effect that contributes significantly to the IRF at short SDD distances: the pixel size of the detector of 0.2 mm × 0.2 mm. At very short distances the pixel size contributes significantly to the FWHM (Table 4 ▸). As the SDD becomes larger the number of illuminated pixels continuously increases and consequently the contribution to the measured FWHM decreases. This indicates that the resolution-limiting factor is the pixel size at short SDD and the divergence originating from the mirrors at larger SDD.

In summary, the IRF of the powder diffraction setup at the ECB originating from the KB or CRL focused beam is reasonable considering the divergence of the beam as well as the pixel size of the XRD 1621 detector. The IRF of the Mar345 is somewhat better than that of the XRD 1621. However, the fact that the XRD 1621 offers a much larger active area (400 mm × 400 mm) and faster readout time generally justifies the usage of this detector over the Mar345 image plate for powder diffraction experiments. Furthermore, at high *q* values the small PSF of the XRD 1621 provides higher-resolution data compared with the Mar345 as pointed out by Daniels & Drakopoulos (2009 ▸). However, for higher-resolution powder diffraction studies one might want to position the XRD 1621 at a SDD larger than 600 mm, at which the IRF becomes flat and is close to the values of the Mar345 located at a SDD of 450 mm.

Good examples of standard powder diffraction experiments at the ECB are studies by Marquardt *et al.* (2011 ▸), Ovsyannikov *et al.* (2013 ▸) and Andrault *et al.* (2014 ▸).

#### 3.4.4. Single-crystal diffraction   

Single-crystal diffraction in the DAC at simultaneously high pressures and high or low temperature is becoming a frequently requested technique at the ECB. Examples for high- and low-temperature single-crystal diffraction experiments are given in §3.5. Single-crystal diffraction images can be collected on the XRD 1621 or the Mar345 image plate in full and stepped rotation mode using an in-house Python script described in detail by Rothkirch *et al.* (2013 ▸). Currently, we can collect diffraction images of ±45° and +45/−30° on the LH and the GP experiment, respectively. For data reduction we employ in-house software (Rothkirch *et al.*, 2013 ▸) enabling step image transformation to the ESPERANTO format of the *CrysAlisPro* software package (*CrysAlisPro* 171.37.34; CrysAlis, Agilent Technologies Ltd., Yarton, Oxfordshire, UK). For calibration of the instrumental parameters within *CrysAlisPro* we collect a full data set of an enstatite single-crystal at the beginning of every beam time. The crystal of enstatite is placed in a symmetric DAC that is equipped with Boehler Almax seats and provides access to reciprocal space of ±32–40° 2θ (depending on the DAC type). At 42.7 keV this results in a sin(θ)/λ_max_ = 0.47 Å^−1^ (SDD 400 mm) offering a powerful tool for single-crystal diffraction. Results of the single-crystal structure analysis of the enstatite (Mg,Fe)SiO_3_ in the DAC under ambient conditions are given in Table 5 ▸, which we use as a standard for calibrating the instrumental parameters within *CrysAlisPro*. Representative examples for single-crystal high-pressure experiments conducted at room temperature are the study on clintonite collected on the Mar345 (Gatta *et al.*, 2012 ▸) and those on talc and mullite (Gatta *et al.*, 2013*a* ▸,*b* ▸) acquired on the XRD 1621. One may note that collection of high-quality single-crystal data of sheet silicates such as clintonite and talc are rather challenging because the *c*-axis (normal to the layers) aligns parallel to the compression axis, restricting the access to two directions in reciprocal space and leaving the *c*-axis usually under-determined. However, because of the short wavelength of 42.7 keV and the relatively large opening in the Boehler Almax DAC, one can collect a reasonable number of Bragg reflections with 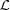 ≠ 0, describing the elastic behavior and the pressure-induced deformation mechanisms at the atomic scale.

#### 3.4.5. Scattering from materials with high degree of atomic disorder   

Scattering on materials with a high degree of atomic disorder such as glasses, melts/liquids and nanocrystalline materials for pair distribution function (PDF) analysis in real space has attracted much attention in the last decade in the field of high-pressure research (Liermann, 2014 ▸, and references therein). Essential for such work is access to a wide *q*-range in reciprocal space to ensure sufficient resolution of the PDF in real space and to be able to calculate accurately the mean atomic density ρ_0_, bond length and coordination numbers. The *q*-range can be increased by using Boehler Almax seats and diamonds as well as performing diffraction experiments at higher energy. At the ECB, PDF studies in the DAC have been performed at 42.7 keV as well as 60 keV with Boehler Almax seats with a total opening of 70°, granting access to a maximum of *q* = 8 Å^−1^ (Sanloup *et al.*, 2013 ▸) and 12 Å^−1^ (Mattern *et al.*, 2013 ▸; Lou *et al.*, 2014 ▸; Rademacher *et al.*, 2014 ▸), respectively. While the diffraction at 60 keV is very demanding because the scattered intensity is rather low especially at high *q* values, this capability is still to be seen as one of the strengths of the ECB. Thus, future beamline upgrades might be geared towards increasing the flux at higher energies by introducing a bent Laue monochromator into the optical train of the beamline. This will significantly increase the overall flux at 60 keV by widening the bandwidth and thus improve counting statistics at large *q* values.

### 3.5. Sample environments   

Besides the laser heating capabilities described earlier (see §3.4.1), the ECB also offers additional static sample environments to generate high pressures at simultaneous high/low temperatures. Of further interest has been the development and implementation of dynamic sample environments that make optimal use of the brightness and detector capabilities of the ECB for fast compression studies. These sample environments have been to a large portion developed in collaboration with the Extreme Conditions Science Infrastructure (ECSI) that is part of the sample environment group at PETRA III (see §4).

#### 3.5.1. Resistive heated DACs   

**Figure 9:**
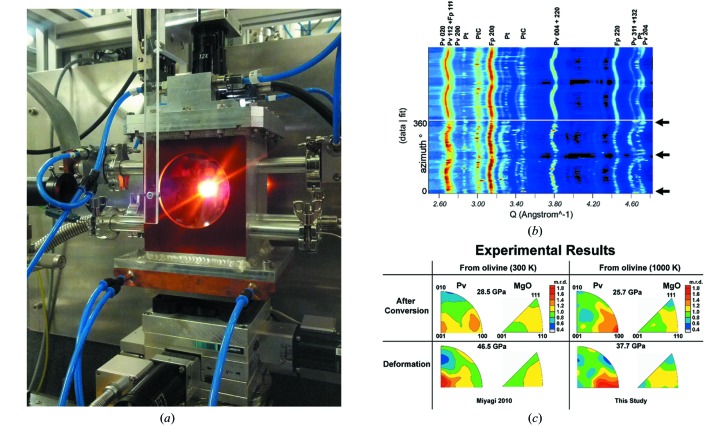
(*a*) The radial diffraction setup at the general purpose experiment of the ECB. In this particular case a sample of ferropericlase was heated to 1125 K (at a certain *P*) and isothermally compressed to 40 GPa (Marquardt *et al.*, 2014 ▸). (*b*) Map plot of the amplitude of X-ray diffraction (that is a stack of X-ray diffraction patterns obtained by integrating two-dimensional XRD images over azimuthal sectors of 5° 2θ). The map plot shows both non-hydrostatic strain (the waviness of the diffraction lines) and preferred orientation (amplitude heterogeneity along the diffraction lines). Simulation shown in the upper part. (*c*) Inverse pole figures of the compression direction of perovskite and ferropericlase at 1000 K and at ambient temperature. The different patterns of the inverse pole figures of the two phases show the features of their preferred orientation at different conditions of *P* and *T* [modified from Speziale *et al.* (2014 ▸)].

Resistive heated DACs are complementing laser heated techniques at temperatures below 2000 K for phase transition as well as thermal equation of state studies. For an overview of resistive heated DAC techniques commonly used at synchrotron facilities one may refer to Liermann (2014 ▸) and references therein. Currently the ECB can provide support for wire heated DACs (*e.g.* Sinogeikin & Bass, 2000 ▸; Sinogeikin *et al.*, 2006 ▸) operating in the lower temperature range from 298 to 700 K and graphite resistive heated DACs for temperatures up to 2000 K (*e.g.* Shen *et al.*, 2007 ▸; Liermann *et al.*, 2009 ▸; Miyagi *et al.*, 2013 ▸; Du *et al.*, 2013 ▸). For these resistive heated DACs the beamline provides a set of three ultra-stable DC power supplies (Agilent Technology, Types 6671A, 6674A, 6675A, with 8 V/220 A, 60 V/35 A, 120 V/18 A) controlled *via* a GPIB and a thermocouple recording system based on a Keithley 3706A with cold junction correction for up to four thermocouples of type K, B or R (other types of thermocouples may be added in due course). Both power supply and thermocouple reading system are implemented in the beamline control software. Besides this basic system for the operation of user-provided resistive heated DACs, in-house research and development has been focusing on the further development of graphite resistive heated DACs for normal and radial diffraction setups in order to make these techniques available to the user community. One of the major improvements has been the construction of vacuum vessels (2 × 10^−4^ mbar) that provide an inert environment to prevent oxidation of the DAC body, the diamonds and the heating circuit (Fig. 9 ▸). In addition to this we have designed and implemented a cooling system for the piston of Mao-Bell DACs used for radial diffraction experiments. The independent cooling of the piston reduces substantially the friction of the piston/cylinder assembly and minimizes seizing during heating, so that one may isothermally compress samples solving a long-standing problem of earlier resistive heated radial diffraction setups (Liermann *et al.*, 2009 ▸; Miyagi *et al.*, 2013 ▸). The new setup has been used successfully to compress ferropericlase isothermally at 825 K up to 70 GPa and at 1125 K up to 40 GPa (Marquardt *et al.*, 2014 ▸). Furthermore, mixtures of ferropericlase and Mg–Fe silicate perovskite have been compressed to 38 GPa at 1000 K (Speziale *et al.*, 2014 ▸). Finally, we have used the graphite resistive heated DAC setup in normal diffraction geometry to collect first single-crystal diffraction patterns at pressures of up to 8 GPa and 1100 K on an amphibole (Liermann *et al.*, 2014 ▸). The single-crystal experiments were performed in an Ar / 2%H_2_ atmosphere. We are currently developing additional vacuum vessels dedicated to powder and single-crystal diffraction experiments in the graphite resistive heated DAC. Results of the improved experimental setups described above together with the data collected during pilot experiments will be published elsewhere.

#### 3.5.2. Cryogenically cooled DACs   

**Figure 10:**
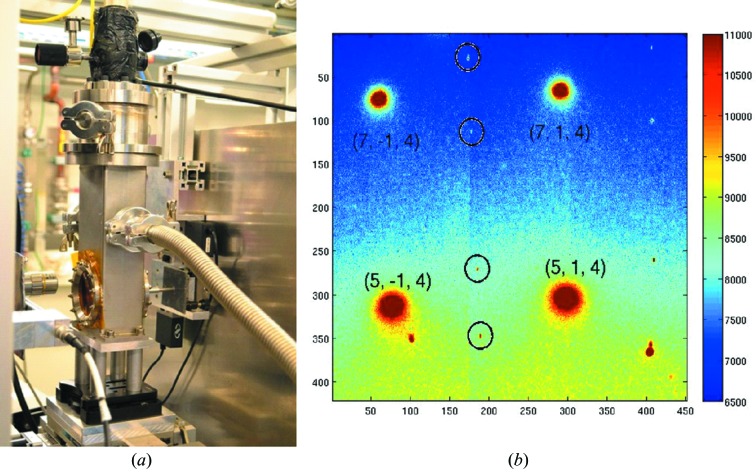
(*a*) Image of the low-temperature setup at the general purpose experiment of the ECB consisting of a modified cold-finger cryostat from Oxford Instruments. (*b*) Diffraction image of the *hk* plane of La_1.67_Sr_0.33_NiO_4_ at *T* = 150 K and *P* = 6.5 GPa, with parts of *l* integrated by sample rotation. The four intense Bragg reflections are labelled with Miller indices (*h, k, l*). The charge stripe order peaks (*h*, 0, 3), with *h* = 4.67, 5.33, 6.67 and 7.33, are marked by circles. Also visible are the corresponding charge order peaks with *k* = 2 on the right-hand side of the image.

There has been a steady demand to combine high pressure with low-temperature conditions, for example for the study of phase stabilities, elastic parameters of ferroelectric materials and low-*Z* elemental solids, such as Li or H. Another field is the study of correlated electron materials where pressure is used as a tool to manipulate the lattice which might have dramatic effects on the system properties. At the moment the ECB is offering a small cold finger cryostat (Optistat CF-V from Oxford Instruments Omicron NanoScience) that is regulated through an Oxford Mercury controller (MERC-TC_2G Mercury ITC). The housing of the cryostat is custom-made and similar to the one used by Guillaume *et al.* (2011 ▸) for the study of the Li phase diagram. It is equipped on the downstream side with a Kapton window and on the upstream side with a transparent polyethylene window offering an opening angle of ±45° on both sides, ideally suited for single-crystal diffraction experiments. Pressure is measured by means of ruby fluorescence through the downstream Kapton window using the online ruby system described in §3.4.2. Copper blocks attached to the cold finger can accept Betsa type MDAC (Membrane Diamond Anvil Cell) (diameter 50 mm) as well as symmetric DACs produced by DESY (diameter 48 mm) located inside and outside of a membrane cup. The accessible temperatures range from ∼45 to 298 K while pressure can be increased and decreased using a membrane control system (Sanchez Technology, Paris, France) for sample temperature above 100 K. The setup has been used to map out phase transitions using both powder and single-crystal diffraction. Recent examples are the powder diffraction studies of the equation of state of Li isotopes (McMahon *et al.*, 2013 ▸; McBride *et al.*, 2014*a* ▸) and the single-crystal study by Hücker *et al.* (2014 ▸) on charge stripe order in La_1.67_Sr_0.33_NiO_4_. Fig. 10 ▸ shows the cryostat setup at the GP experiment as well as a diffraction image collected at 150 K of stripe ordered La_1.67_Sr_0.33_NiO_4_ at 6.5 GPa. The positions of the intense fundamental reflections are indicated. Despite being three orders of magnitude less intense, the superlattice reflections from charge stripe order can be well identified as indicated by circles in Fig. 10(*b*) ▸. More detailed reports on the above-mentioned experiments will be published elsewhere. In order to further extend the low-temperature high-pressure capabilities of the ECB and PETRA III in general, the ECSI is designing a He flow cryostat that should be able to reach temperatures as low as 2–4 K at which point pressure in the DAC has to be increased through a gear box device from the outside. This cryostat should also have single-crystal diffraction capabilities, *i.e.* have opening windows of ±35° and offer one rotational degree of freedom over the same angular range.

#### 3.5.3. Fast compression DACs   

**Figure 11:**
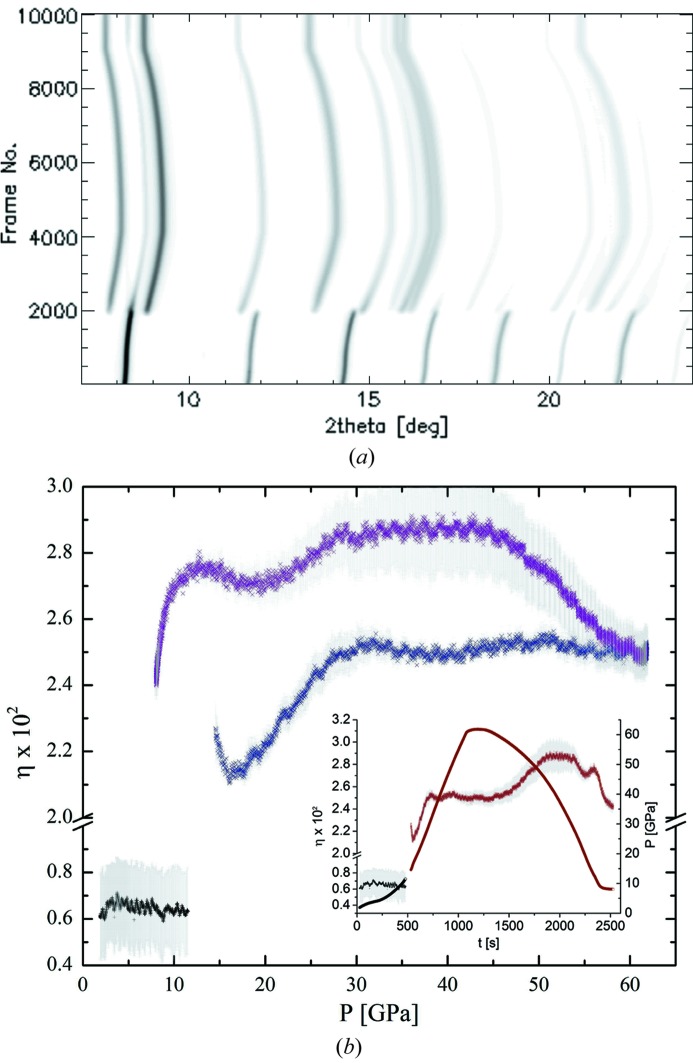
(*a*) Contour plot and (*b*) micro-strain in α-Fe (black plus signs) and ∊-Fe (blue crosses: compression; purple crosses: decompression) as a function of pressure during a relatively slow compression experiment (0.09 GPa s^−1^). The grey areas in (*b*) denote errors of the regression fits to the FWHM data (the standard error of the linear fit is represented here by a plus–minus error bar). Reproduced from Konôpková *et al.* (2015 ▸).

**Figure 12:**
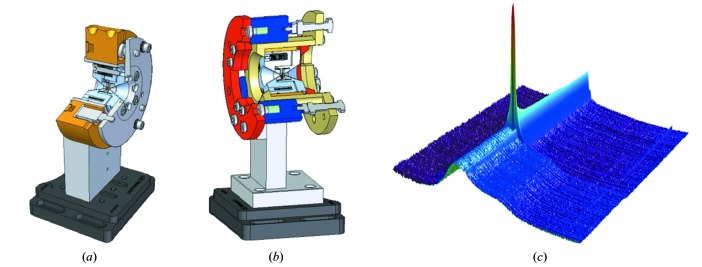
Symmetric piston cylinder type DAC included in (*a*) a dynamic decompression (Wittich, 2013 ▸) and (*b*) dynamic compression setup developed at the ECB. (*c*) Integrated one-dimensional diffraction patterns of liquid gallium on pressure increase using the dynamic DAC. Pressure is increased at a rate of 1 GPa s^−1^ and two-dimensional diffraction images were collected every 67 ms using an XRD 1621 detector. One may clearly see the growth of a solid crystal of Ga-III from the liquid.

One of the focus areas of the ECB is the development and the implementation of time-resolved diffraction experiments that can be used to investigate the effect of different compression rates on the occurrence of phase transitions as well as their influence on the elastic/plastic behavior of materials. There are two techniques that have been used to create such fast compression rates, the membrane-driven DAC (mDAC) or a dynamically driven DAC (dDAC; Evans *et al.*, 2007 ▸). The mDAC has recently been used to create very rapid compressions with a pressure jump controller (Velisavljevic *et al.*, 2012 ▸, 2014 ▸) up to 400 GPa s^−1^ whereas the dDAC has been used up to compression rates of 500 GPa s^−1^ (Evans *et al.*, 2007 ▸). The two techniques differ in that the mDAC can cover at the moment a very large pressure interval, *e.g.* from 0.0001 to 80 GPa, however, with limited control on the shape of the pressure curve. In contrast, the dDAC creates high compression rates in only a very limited pressure range of 10–20 GPa while enabling a close control on the shape of the compression curve. Thus, both techniques are complimentary but limited to maximum strain rates of 10^2^ s^−1^. Nevertheless, they are the techniques that can be used to bridge the gap between static and highly dynamic compression conditions reached during flyer plate experiments with strain rates of the order of 10^4^ s^−1^ and gas gun or laser shock compression experiments with rates >10^6^ s^−1^ (Forbes, 2012 ▸). The challenge for any of these experiments is not so much creating fast compression (pumping) but rather to find an adequate probe, *e.g.* for structural analysis *via* X-ray diffraction. Shock compression diffraction experiments have been conducted at third-generation sources [*e.g.* Turneaure *et al.*, 2009 ▸; Turneaure & Gupta, 2009 ▸; Gupta *et al.*, 2012 ▸; or see Dynamic Compression Sector (DCS) at the APS, Chicago, USA], but they are probably even better suited for instruments such as the MEC (Matter at Extreme Conditions; Nagler *et al.*, 2015 ▸) at the Linear Coherent Light Source (LCLS) or the upcoming HED (High Energy Density) instrument at the European XFEL because of the increased brilliance and the tenth of femtosecond pulse length that enable very short single-shot diffraction experiments. Fast compression experiments in the DAC (mDAC and dDAC) are still manageable at a ‘high resolution’ diffraction beamline such as the ECB but could also benefit from the pulse train structure of the European XFEL (4.5 MHz separation within the pulse train). At the ECB we have been conducting both fast diffraction experiments in the mDAC and the dDAC. In fact, we have been developing, in collaboration with the Lawrence Livermore National Laboratory (LLNL) high-pressure group, a dDAC setup that can be used with symmetric piston cylinder type DACs (Wittich, 2013 ▸). The limiting factor for X-ray diffraction experiments in the mDAC and dDAC is currently the amount of flux available after focusing as well as the readout speed of the detector. Three different types of detectors have been used for fast compression experiments at the ECB: the XRD 1621 (15 Hz at both 25.6 and 42.7 keV), the PILATUS 300K as well as the PILATUS 1M from the loan pool of the DESY detector group (30 Hz upon reading a single tile, 25.6 keV) and the new GaAs-based LAMBDA prototype detector (1 kHz at 42.7 keV; Pennicard *et al.*, 2013 ▸). All three detectors have been used successfully to collect diffraction patterns at their maximum frame rate with the flux available at the ECB after focusing. Each detector has its advantages and limitations and should be chosen carefully depending on the nature of the compression experiments. The XRD 1621 is well suited for diffraction experiments at 42.7 keV when one likes to see entire diffraction rings to be able to visualize potential texture effects, *e.g.* when studying metals like iron as described by Konôpková *et al.* (2015 ▸). In that study the authors compress iron at room temperature under quasi- and non-hydrostatic conditions to systematically study the phase-transition pressure and micro-stresses as a function of compression rate using the mDAC and reached maximal pressures and compression rates of 70 GPa and 4.1 GPa s^−1^ (strain rates of 10^−2^ s^−1^; Fig. 11 ▸), respectively. While the study did not reveal any change of transition pressure when going from α-Fe to ∊-Fe within the range of the investigated compression rate, it did produce some interesting insights into the overall strain field encountered during fast compression in the mDAC. These results might hint at the advantages and limitations of fast compression experiments in the DAC and will provide important information for future experiments. Similar fast compression studies in the mDAC have been performed on α-quartz and its high-pressure polymorphs (Carl *et al.*, 2014 ▸) and elemental gallium (McBride *et al.*, 2014*b* ▸) as well as on Zn metal (Velisavljevic *et al.*, 2014 ▸) and organic compounds such as *tert*-butyl acetylene [(CH_3_)_3_—C=CH] (Velisavljevic *et al.*, 2012 ▸). PILATUS detectors were used in conjunction with the dDAC setup developed by the LLNL high-pressure group (Evans *et al.*, 2007 ▸). Examples of their work are illustrated in the dynamic compression studies on ice (Chen *et al.*, 2014*a* ▸) and krypton (Chen *et al.*, 2014*b* ▸). The new dDAC setup developed at the ECB (Fig. 12 ▸; Wittich, 2013 ▸) is still being commissioned but has been used during a recent beam time for a dynamic compression study on Ga with the prototype of the GaAs type LAMBDA detector to collect diffraction images at a rate of 1 kHz (McBride *et al.*, 2014*b* ▸). Unfortunately, the GaAs LAMBDA prototype detector has only a very small active area of 4.2 cm (H) × 2.8 cm (V) so that one can cover only a very limited amount of reciprocal space. In the future PETRA III will invest in the further development of detectors such as the GaAs LAMBDA detector to conduct more effectively time-resolved X-ray scattering experiments at a larger *q*-range.

### 3.6. Summary of beamline capabilities and outlook   

The Extreme Conditions Beamline (ECB) P02.2 is a versatile micro-diffraction beamline optimized for studies at extreme conditions of high pressure and simultaneous high and low temperatures. The different research focus areas that arise from the technical capabilities of the beamline can be grouped into three categories:

(*a*) Very small beam: enables static powder and single-crystal diffraction experiments at very high pressures and high/low temperatures.

(*b*) High-brilliance and fast detectors: provide tools for dynamic powder diffraction at high pressures and high/low temperatures.

(*c*) High-energy X-rays: possibility to conduct total scattering and PDF studies on powders and non-crystalline materials.

In particular, the high brilliance at high energies (short wavelength) makes the ECB unique among other high-pressure beamlines so that future developments at the beamline could focus on further strengthening these capabilities. Such projects could be the implementation of double focusing in order to increase the beam acceptance at the KB mirror system or the use of the full third and fifth harmonic from the undulator; both implementations will increase flux at the sample position and may also reduce exposures during time-resolved studies of low-*Z* materials. At the same time developments of faster and more sensitive high-energy area detectors will further improve our capabilities to conduct time-resolved diffraction studies. Finally, the implementation of a graded Laue monochromator could increase the flux at 60 keV and provide improved conditions to conduct total scattering and pair distribution function studies. Some of these ideas are already on the list for the beamline upgrade for the next few years; some will require more detailed considerations and the allocation of necessary funds.

## 4. Extreme Conditions Science Infrastructure   

The ECSI was developed with the knowledge that extreme conditions research at high pressure and simultaneous high and low temperature will flourish if the sample environment group at PETRA III supports this type of research at other beamlines. Thus, the idea developed to share preparation laboratories and other supporting facilities (such as offline laser systems) between the sample environment group and the ECB. Today there are 2.5 preparation laboratories and a large laser laboratory with six laser systems that are jointly used and maintained by the sample environment group and the ECB and that make up the core of the ECSI. Below we provide a small overview of the different systems implemented.

### 4.1. Preparation laboratories   

Two preparation laboratories are available to users of the ECSI and ECB, one laboratory is dedicated to the assembly, alignment and loading of normal DACs and another laboratory for the preparation of resistive heated DACs. Both laboratories are equipped with stereo microscopes (Leica M165 C) and all necessary tools for assembly and loading of the specific DACs. In addition, the second laboratory houses a gas loader (Sanchez Technology, Paris, France) for the high-pressure loading of He, Ne, Ar and N_2_. Currently, symmetric piston cylinder type, four pin and Betsa type DACs can be loaded in this apparatus, while additional DAC types can be implemented in the future depending on demand. Loading of a DAC takes approximately 4 h since decompression is performed *via* a membrane attached to the DAC. Mechanical-driven DACs can be closed outside the gas loader after closing them with the membrane within a cup. At the moment we are implementing an additional ruby system for pressure measurements during closing of the DACs with the membrane. The preparation laboratory is also equipped with a fume hood to load DACs with a Be gasket, a capability desired for radial X-ray diffraction and X-ray spectroscopy experiments.

### 4.2. Laser laboratories   

The laser laboratory of the ECSI is located in close proximity to the ECB. It consists of an area that houses several class 1 laser systems such as the offline ruby system, the offline Raman system and an excimer laser micro-machining system for shaping diamonds as well as drilling metal and ceramic gaskets. This system was implemented by the University of Frankfurt (PI: B. Winkler). The laser laboratory also contains a separate class 4 laser enclosure that houses an offline Brillouin scattering system that was funded and built by the GFZ, Potsdam (Speziale *et al.*, 2013 ▸), an offline laser heating system that was in part developed in collaboration with the University of Frankfurt (PI: B. Winkler) and a portable laser system for beamline P01 that was built by the University of Dortmund (PI: C. Sternemann and M. Tolan). Below we will describe briefly the offline ruby and Raman system.

#### 4.2.1. Offline ruby system   

**Figure 13:**
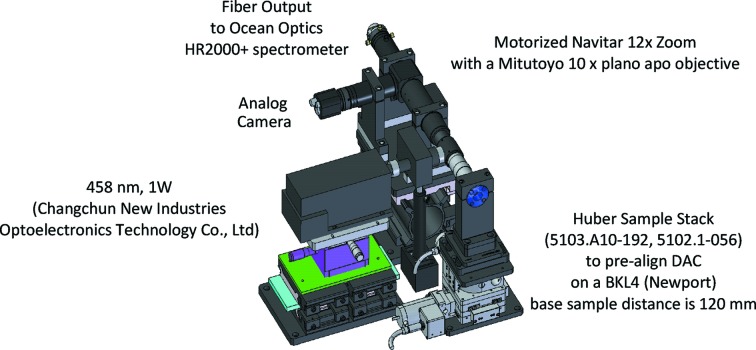
Three-dimensional model of the offline ruby system located in the laser laboratory of the ECSI.

The offline ruby system was built to pre-align DACs for X-ray measurements at the ECB or determine pressure *via* ruby fluorescence (Mao *et al.*, 1978 ▸) prior to beam time at the ECB. The system is also used to enable ruby measurement for other beamlines at PETRA III not equipped with pressure measurement capabilities. The system consists of a motorized 12× zoom system (Navitar; Fig. 13 ▸) equipped with a laser injection port and a 10× Plano Apo objective (Mitutoyo) with 30.5 mm working distance. We modified the system by replacing the prism in the laser injection port with a ‘Bright Light 442 nm Laser Dichroic Beamsplitter’ in order to feed in the 457.9 nm, 1 W laser (Changchun New Industries Optoelectronics Technology) and installing an Aspheric Fiber Port (Thorlabs) to guide the ruby fluorescence signal *via* a 50 µm patch cable to the HR-2000 spectrometer (Ocean Optics). Ruby spheres in the DAC may be aligned to the laser system through a small XYZ sample stack (Huber). The DAC holders are positioned on the XYZ stack *via* the BKL4 kinematic mount (Newport). The distance from the base of the BKL4 to the sample position is normalized to 120 mm, identical to the distances on the stacks of the ECB.

#### 4.2.2. Offline Raman system   

**Figure 14:**
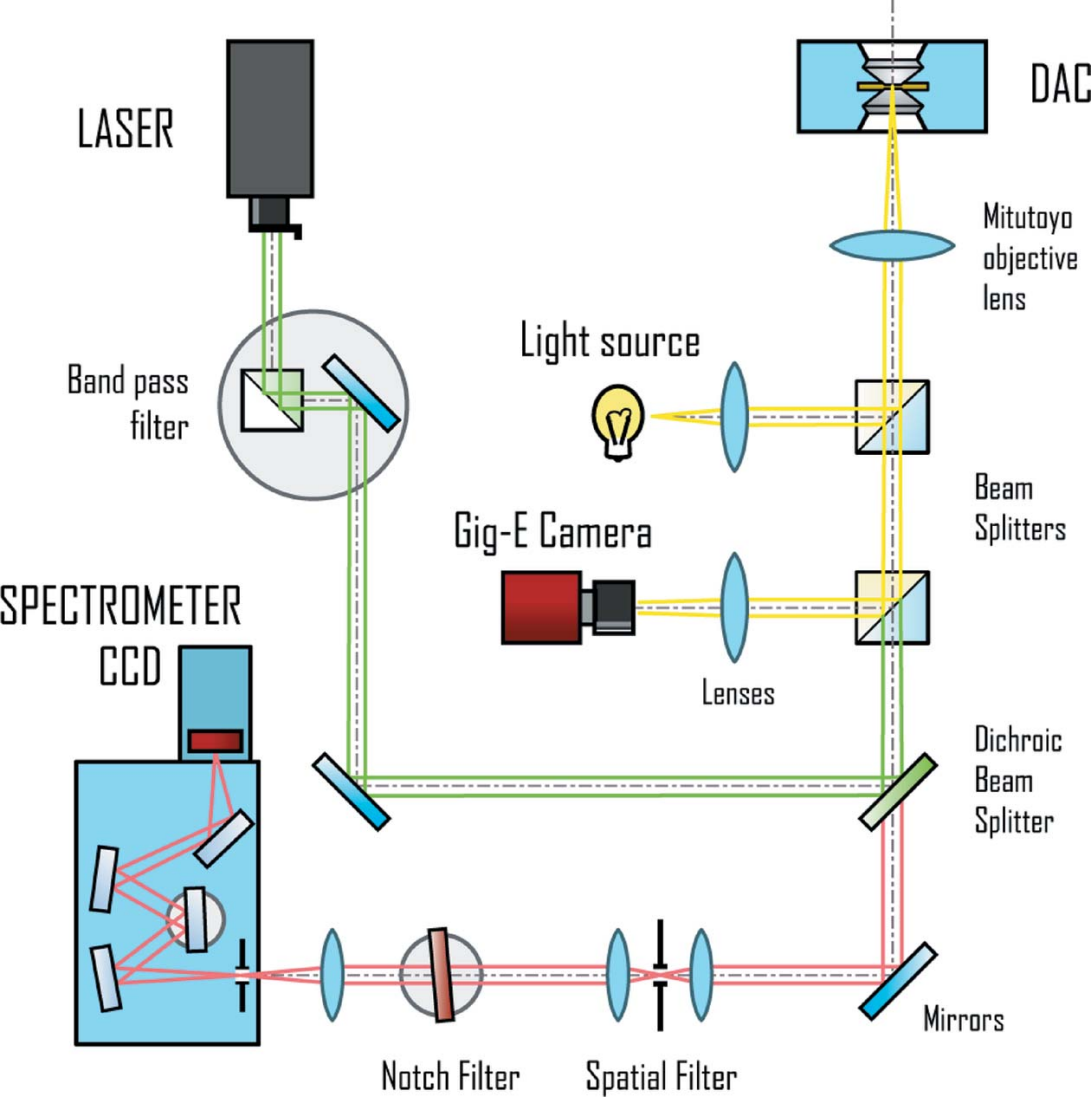
Optical path of the modular offline Raman system located within the class 1 laser laboratory of the ECSI.

Raman spectroscopy is an essential part of any beamline that serves the high-pressure community to estimate pressure, *e.g.* *via* the distortion of the Raman diamond line at 1333 cm^−1^ (*e.g.* Akahama & Kawamura, 2010 ▸), to identify phases or collect additional structural information. The ECB is in the process of installing/commissioning online Raman systems; however, their capabilities will always be limited so a significant amount of effort was spent designing and building a modular offline system that can be customized to a certain degree to the user’s needs. At the moment the system consists of two laser beam paths (Fig. 14 ▸), one powered with a 532 nm, 300 mW laser (Laserglow Technology) and the other with a 458 nm, 300 mW laser (Melles Griot). Both paths contain specific bandpass filters and Super-Notch-plus filters (Kaiser Optics). Lasers are guided into the reflecting beam path of the Raman signal *via* RazorEdge^®^ filters at 532 and 458 nm (Semrock). Raman signal is analyzed with a Shamrock SR-500i-A spectrometer and a DU940N-BV CCD (13.5 µm × 13.5 µm pixel and 2048 × 521 pixels, ANDOR Technology).

## 5. Conclusion   

The Extreme Conditions Beamline P02.2 offers micro X-ray diffraction capabilities well suited for materials research, physics, chemistry and geoscience at simultaneously high pressure and high/low temperatures in a DAC. The brilliance of the beamline combined with a very small and high-energy X-ray beam make the ECB the ideal instrument for studying powders and single crystals at multi-megabar pressures and simultaneous high and low temperatures to shed light on questions relevant to geophysics and high-pressure physics. The high-energy X-ray diffraction capabilities at 42.7 and 60 keV are well suited for the study of materials with a high degree of atomic disorder, a growing field in high-pressure physics. Of particular interest is the use of the brilliance of the ECB to conduct fast compression experiments in dynamically compressed DACs, enabling millisecond exposure times. The experimental capabilities of the ECB are complemented by the versatile Extreme Conditions Science Infrastructure that also serves high-pressure and simultaneous high- and low-temperature research at other beamlines of PETRA III.

## Supplementary Material

Crystal structure: contains datablock(s) I. DOI: 10.1107/S1600577515005937/ie5136sup1.cif

Structure factors: contains datablock(s) global, I. DOI: 10.1107/S1600577515005937/ie5136sup2.hkl

Structure factors: contains datablock(s) global, I. DOI: 10.1107/S1600577515005937/ie5136sup3.hkl

## Figures and Tables

**Table 1:** Machine parameters of the PETRAIII storage ring (from Bacher *et al.*, 2007 ▸) and parameters of the planar undulator U23 for 100mA (from Barthelmess, 2008 ▸)

Machine parameters			Insertion device parameters	
Energy	6 GeV		Device	U23
Circumference	2304 m		Minimum magnetic gap	9.5 mm
Harmonic number	3840		Period length _U_	23 mm
HF	500 MHz		Length *L*	2 m
Horizontal emittance	1 nmrad		Peak field *B*_0_	0.61 T
Coupling factor	1%		Deflection parameter	1.3
Beam current	100 mA		First harmonic *E*_1_	8.0 keV
No. of bunches	960 or 40		Total power *P*_tot_	1.7 kW
_*x*_	20.12 m		On-axis power density	71 kW mrad^2^
_*y*_	2.36 m		Power in 1mm 1mm at 40m	44 W
			High- source size (10keV)	140 m (H) 5.6 m (V)
			High- source divergence (10keV)	7.9 rad (H) 4.1 rad (V)

**Table 2:** Upper boundary for the energy resolution calculated from the FWHM of Si(111) and the Si(311) crystal rocking curves of the second crystal of the DCM at 42.40 and 59.82, 76.95keV, respectively Rocking curves were determined by scanning the second monochromator crystal against the first crystal and thus giving an upper bound of the energy resolution.

Crystal	Energy (keV)	Theoretical rocking curve (FWHM rad^1^)	Measured rocking curve (FWHM rad^1^)	*E*/*E* (meas) (FWHM)
Si(111)	42.40	8.38	9.79 (9)	2.1 10^4^
Si(311)	59.82	2.38	3.99 (7)	6.3 10^5^
Si(311)	76.95	1.57	5.3 (1)	1.07 10^4^

**Table 3:** Focusing available at the ECB for different energies and focusing devices

Energy (keV)	Focus device	Beam size† at sample position (FWHM m^2^)	Divergence‡ (mrad)	Flux at sample position (photons s^1^)
25.6	KB	2 (H) 2 (V)	1.2 (H) 0.63 (V)	9.3 10^11^ §
42.7	KB	2 (H) 2 (V)	1.2 (H) 0.63 (V)	6 10^10^ §
25.6	40 CRL (Be)	8 (H) 2 (V)	0.31 (H) 0.31 (V)	2 10^11^ ¶
42.7	121 CRL (Be)	8 (H) 2 (V)	0.25 (H) 0.26 (V)	5 10^10^ ¶
60.0	139 CRL (Al)	8 (H) 2 (V)	0.16 (H) 0.16 (V)	n/d

† Measured values representing the smallest average focus. Larger focal size is possible.

‡ Calculated values based on the distance from the center of the optical device to the sample position and the effective acceptance of the CRL stack.

§ Measurements performed without pinhole.

¶ Measurements performed with a 40m pinhole.

**Table 4:** Theoretically calculated contributions to the IRF Values are normalized to 42.4keV (0.292416) at a 2 angle of the Si(111) reflection (2.6728). The total of the value of the IRF was calculated assuming a predominantly Gaussian shape for the diffraction peaks, *i.e.*_total_ = [(_i_)^2^]^1/2^.

Origin of contribution	Comment	(H) ()	(V) ()	*E*/*E* (H)	*E*/*E* (V)
Monochromator (energy)		0.0006	0.0006	0.00021	0.00021
Focusing (divergence)	1.2 mrad (H) 0.63 mrad (V)	0.0709	0.0355	0.02651	0.01325
Sample geometry		0.0007	0.0007	0.00026	0.00026
Pixel size broadening, SDD = 350	1.1 (0.2mm 0.2mm)	0.0327	0.0327	0.01224	0.01224
Pixel size broadening, SDD = 1000	1.1 (0.2mm 0.2mm)	0.0115	0.0115	0.00428	0.00428
Total (SDD 350)		0.0781	0.0482	0.0292	0.01805
Total (SDD 1000)		0.0718	0.0372	0.0269	0.01393

**Table:** (*x*, *y*, *z*) and *U*_ani_ correspond to coordinates of the elements and atomic displacement parameters in harmonic model, respectively. *U*_ani_ of Fe and Mg occupying same crystallographic sites was refined as a single variable. We also determined element-specific *U*_ani_ variables corresponding to each of the Si and O atoms. Energy is 42.8keV, and distance to detector is 400mm. Number of unique reflections is 1356. Refinement of integrated intensities was conducted with *Jana2006* (Petrcek *et al.*, 2014 ▸). The refinement converged with *R*-factor *R*_all_ = 4.98%. Prior to integration of diffraction intensities, the same single-crystal was used in Agilent *CrysAlisPro* for instrument model refinement. Site labeling and coordinates are given according to the generally accepted specification for orthoferrosilites (Burnham, 1966 ▸). Occ.: occupancy; Mult.: site multiplicity. Superscripts O and T indicate orthorhombic and tetrahedral atomic environments, respectively.

Lattice parameters: *a* = 18.2457(9), *b* = 8.8201(7), *c* = 5.1875(2)

**Table:** 

Structure parameters							
Site	Atom	Mult.	Occ.	*x*	*y*	*z*	*U*_iso_
M1^O^	Mg	8c	0.982 (3)	0.37593 (3)	0.65364 (8)	0.8648 (1)	0.0050 (2)
	Fe	8c	0.018 (3)	0.37593 (3)	0.65364 (8)	0.8648 (1)	0.0050 (2)
M2^O^	Mg	8c	0.963 (3)	0.37738 (3)	0.48573 (8)	0.3577 (1)	0.0069 (2)
	Fe	8c	0.037 (3)	0.37738 (3)	0.48573 (8)	0.3577 (1)	0.0069 (2)
A^T^	Si	8c	1	0.27157 (2)	0.34169 (6)	0.0490 (1)	0.0040 (1)
B^T^	Si	8c	1	0.473497 (19)	0.33726 (6)	0.7997 (1)	0.0041 (1)
A^T^	O	8c	1	0.18323 (5)	0.33988 (16)	0.0342 (2)	0.0054 (3)
	O	8c	1	0.31082 (6)	0.50248 (17)	0.0424 (2)	0.0070 (4)
	O	8c	1	0.30304 (6)	0.22298 (16)	0.1699 (2)	0.0065 (3)
B^T^	O	8c	1	0.56271 (5)	0.33975 (16)	0.8013 (2)	0.0053 (3)
	O	8c	1	0.43267 (6)	0.48342 (17)	0.6890 (2)	0.0071 (4)
	O	8c	1	0.44730 (6)	0.19387 (17)	0.6056 (2)	0.0065 (4)

**Table:** 

Atomic displacement parameters							
		*U*_11_	*U*_22_	*U*_33_	*U*_12_	*U*_13_	*U*_23_
M1^O^	Mg	0.0052 (3)	0.0054 (4)	0.0044 (2)	0.0001 (2)	0.0004 (1)	0.0001 (2)
	Fe	0.0052 (3)	0.0054 (4)	0.0044 (2)	0.0001 (2)	0.0004 (1)	0.0001 (2)
M2^O^	Mg	0.0073 (3)	0.0073 (4)	0.0061 (2)	0.0011 (2)	0.0018 (1)	0.0007 (2)
	Fe	0.0073 (3)	0.0073 (4)	0.0061 (2)	0.0011 (2)	0.0018 (1)	0.0007 (2)
A^T^	Si	0.0034 (2)	0.0044 (3)	0.0041 (1)	0.0004 (1)	0.0000 (8)	0.0003 (1)
B^T^	Si	0.0033 (2)	0.0045 (3)	0.0046 (1)	0.0002 (1)	0.0005 (1)	0.0002 (1)
A^T^	O	0.0032 (4)	0.0070 (8)	0.0061 (3)	0.0003 (3)	0.0004 (2)	0.0002 (3)
	O	0.0066 (5)	0.0069 (9)	0.0075 (3)	0.0026 (3)	0.0007 (2)	0.0009 (3)
	O	0.0058 (5)	0.0082 (8)	0.0054 (3)	0.0013 (3)	0.0004 (2)	0.0029 (3)
B^T^	O	0.0044 (4)	0.0054 (8)	0.0060 (3)	0.0004 (3)	0.0003 (2)	0.0002 (3)
	O	0.0070 (5)	0.0078 (9)	0.0064 (3)	0.0029 (3)	0.0007 (2)	0.0002 (3)
	O	0.0057 (5)	0.0071 (9)	0.0067 (3)	0.0008 (3)	0.0005 (2)	0.0026 (3)
